# Optimisation and Characterisation of Anti-Fouling Ternary SAM Layers for Impedance-Based Aptasensors

**DOI:** 10.3390/s151025015

**Published:** 2015-09-29

**Authors:** Anna Miodek, Edward M. Regan, Nikhil Bhalla, Neal A.E. Hopkins, Sarah A. Goodchild, Pedro Estrela

**Affiliations:** 1 Department of Electronic and Electrical Engineering, University of Bath, Bath BA2 7AY, UK; E-Mails: ed_regan@hotmail.com (E.M.R.); n.bhalla@bath.ac.uk (N.B.); 2 Defence Science and Technology Laboratory, Porton Down, Salisbury, SP4 0JQ, UK; E-Mails: nahopkins@dstl.gov.uk (N.A.E.H.); sagoodchild@dstl.gov.uk (S.A.G.)

**Keywords:** biosensor, DNA aptamers, self-assembled monolayers, antifouling, screen printed electrodes, electrochemical detection, Thrombin

## Abstract

An aptasensor with enhanced anti-fouling properties has been developed. As a case study, the aptasensor was designed with specificity for human thrombin. The sensing platform was developed on screen printed electrodes and is composed of a self-assembled monolayer made from a ternary mixture of 15-base thiolated DNA aptamers specific for human thrombin co-immobilised with 1,6-hexanedithiol (HDT) and further passivated with 1-mercapto-6-hexanol (MCH). HDT binds to the surface by two of its thiol groups forming alkyl chain bridges and this architecture protects from non-specific attachment of molecules to the electrode surface. Using Electrochemical Impedance Spectroscopy (EIS), the aptasensor is able to detect human thrombin as variations in charge transfer resistance (*R*_ct_) upon protein binding. After exposure to a high concentration of non-specific Bovine Serum Albumin (BSA) solution, no changes in the *R_ct_* value were observed, highlighting the bio-fouling resistance of the surface generated. In this paper, we present the optimisation and characterisation of the aptasensor based on the ternary self-assembled monolayer (SAM) layer. We show that anti-fouling properties depend on the type of gold surface used for biosensor construction, which was also confirmed by contact angle measurements. We further studied the ratio between aptamers and HDT, which can determine the specificity and selectivity of the sensing layer. We also report the influence of buffer pH and temperature used for incubation of electrodes with proteins on detection and anti-fouling properties. Finally, the stability of the aptasensor was studied by storage of modified electrodes for up to 28 days in different buffers and atmospheric conditions. Aptasensors based on ternary SAM layers are highly promising for clinical applications for detection of a range of proteins in real biological samples.

## 1. Introduction

There is a substantial need for the development of biosensors capable of sensitive and selective detection, that are also low cost, easy to use and have the possibility of being integrated into portable devices for point-of-care use with clinical or environmental samples. Electrochemical biosensors are some of the most promising platforms with the potential to achieve these goals and have been used to detect a number of analytes. These include, e.g., (1) disease biomarkers and pathogenic proteins in serum [1,2], human plasma [3,4] or urine [5]; (2) Nucleic acids such as DNA samples from polymerase chain reaction (PCR) products [6,7] and microRNAs [2,8]; (3) microorganisms in seawater medium [9] and tap water [10]; (4) toxins in food samples [11]; and (5) drugs levels in human serum [12,13,14].

The applications of biosensors for complex samples, whether they be environmentally or clinically derived, are, however, limited by their fouling properties due to non-specific binding of background materials to the measurement surfaces. One challenge to be overcome in construction of biosensors with broad potential for exploitation is therefore the elimination of this non-specific adsorption of molecules to the electrode surface. Different strategies have previously been developed to prevent non-specific binding. The most common method has been to generate binding resistant layers through manipulation of either the components or the construction of the self-assembled monolayer. This can be achieved by co-immobilisation of bioreceptors with alkanethiols of varying lengths of alkyl chains such as 1-mercapto-6-hexanol (MCH) [15] or 11-mercapto-1-undecanol (MCU) [16], which simultaneously can act as spacers to avoid steric hindrances. Modifications of the self-assembled monolayer (SAM) components can also be performed. For example, prostate specific antigen (PSA) aptamers were immobilised on a SAM layer containing a thiolated sulfo-betaine antifouling agent [17]. Sulfo-betaine moiety prevented any significant non-specific binding of the control protein human serum albumin (HSA) as compared to high non-specific binding with MCH-based sensors.

Poly (ethylene glycol) (PEG) and its derivatives are the most commonly used anti-fouling materials for biosensor construction. For example, in a study used for detecting embryonic stem cell lysate with surface plasmon resonance imaging, the surface was modified with thiolated PEG chains or three dimensional polymers containing PEG chains [18]. These surfaces combined with a good blocking solution, such as amine terminated PEG, and adequate running buffer supplemented with detergent exhibited greatly decreased non-specific binding. Thiolated oligomers with di-(ethylene glycol) [19] or tri-(ethylene glycol) (OEG) [20] groups have also been used to generate stable non-fouling background and OEG monomers constructed into polymer “brushes” have been shown to be resistant to the adsorption of proteins from complex media such as undiluted foetal bovine serum [21]. This class of surface has been successfully used for detection of analytes associated with viral infection in clinical samples [22]. Among other polymers, poly-(ethyleneoxide) (PEO) derivatives were able to protect the surface from non-specific adsorption after exposition on buffers containing either 0.1% bovine serum albumin or 1% goat serum [23]. Low fouling background can be also obtained using zwitterionic polymers containing groups such as phosphorylcholine (PC), sulfobetaine (SB), and carboxybetaine (CB). These materials constitute an interesting alternative to PEG-based surfaces protecting from non-specific interactions even in complex media [24,25,26,27]. In addition, diblock copolymer “brushes” composed of OEG and polyCB were reported to improve fouling properties of biosensors and minimalize adsorption from blood plasma [28]. Ionic liquid self-assembled monolayers have been investigated for lower non-specific binding. Ratel *et al.* developed a SAM monolayer based on ionic liquids with low fouling in crude serum, to a level equivalent to PEG [29]. However, the majority of these surface types are limited in their application to optical sensors based on the surface plasmon resonance technique, because of high molecular weight, which results in detrimental effects on electrochemical measurements and blocking effects on charge transfer.

Much fewer examples exist concerning the development of anti-fouling materials compatible with electrochemical sensing in clinical samples or complex media [30]. Among such materials, hyperbranched polyester microspheres [31], hydrophilic polymer carboxymethyl-PEG-carboxymethyl (CM-PEG-CM) [32] or zwitterionic surfaces [33,34] have been evaluated. However, due to the high molecular weight of these compounds and potential impacts on electrochemical transfer reactions, these materials still present serious limitations in electrochemical sensing applications.

Recently Campuzano *et al.* demonstrated the anti-fouling properties of a ternary self-assembled monolayer for construction of electrochemical DNA sensors [35]. This simple layer, composed of co-immobilised thiolated DNA probe with 1,6-hexanedithiol (HDT) and saturated with 1-mercapto-6-hexanol (MCH), was used for detection of DNA targets in serum and urine samples. The study demonstrated improved signal-to-noise ratio with high hybridisation efficiency and detection limit on the attomolar level. The improved antifouling properties of this surface was attributed to the favourable surface architecture by the presence of HDT which may adopt a horizontal configuration, acting as a bridge and providing significantly higher resistance to nonspecific adsorption of nucleic acids and proteins. The authors showed the advantage of the ternary SAM over the commonly used binary DNA/MCH SAM, which presents poor anti-fouling properties (as also seen in our previous studies [17]). This can be due to the hydroxyl groups that can electrostatically bind to chemical groups of proteins. Furthermore, the use of a ternary SAM surface improves reproducibility comparing to a DNA/MCH layer and it was also proven that can extend storage stability of a genosensor [36].

The aim of this work is the application of the ternary SAM layer developed by Campuzano *et al.* in the construction of impedimetric DNA aptamer-based biosensors. As a case study, the anti-fouling properties of the ternary SAM layer and analytical properties of this aptasensor were assessed with low molecular weight thrombin aptamers specific for thrombin from human serum. The G-quadruplex structure of this aptamer system was considered likely to affect the bridge architecture obtained by using HDT, and as such different conditions of biosensor construction were studied to ensure a well performing system could be identified. The system was therefore optimised in terms of surface construction performing an empirical assessment of the ideal ratio of aptamer to HDT within the ternary mixture. The results clearly show the influence of the quantity of aptamer immobilised on the surface in relation to non-specific interactions. The influence of buffer pH and temperature was also studied. The biosensor was developed on Screen Printed Electrodes (SPE) and contrary to classic three-compartment cells an improvement of signal-to-noise ratio was observed as compared to standard gold electrodes. Finally the storage stability of the biosensor and influence of different storage conditions on anti-fouling properties and performance were studied.

## 2. Experimental Section

### 2.1. Reagents

Thrombin (Th) from human plasma was purchased from Sigma-Aldrich. Thiol-modified aptamer specific for Th (ThA aptamer) with dT_3_ and six carbon spacer on 5' phosphoryl terminus (5'-HS-C_6_-dT_3_-GGT TGG TGT GGT TGG-3') were provided by Sigma-Aldrich (HPLC purified) and dissolved in TE buffer consisting of 10 mM tris (hydroxymethyl) aminomethane (Tris) and 1 mM ethylene di-amine tetra-acetic acid (EDTA) at pH 7.6 in ultra-pure water. 1,6-hexanedithiol (96%), 6-mercapto-1-hexanol (97%), potassium phosphate monobasic solution (1 M), potassium phosphate dibasic solution (1 M), potassium sulphate, potassium hexacyanoferrate (III), potassium hexacyanoferrate (II) trihydrate, ethylenediaminetetraacetic acid (EDTA, 0.5 M) and Tris were provided from Sigma Aldrich. All buffers solutions were prepared using 18.2 MΩ cm ultra-pure water (Millipore, Billerica, MA, USA) with a Pyrogard filter (Millipore) to remove nucleases, stored at 4 °C until use and filtered by 0.22 µm syringe membranes prior use. Non-specific interactions were studied using Bovine Serum Albumin (BSA) (Sigma-Aldrich).

### 2.2. Instrumentation

*Electrochemical Impedance Spectroscopy* (EIS) measurements were performed using a μAutoLab III/FRA2 potentiostat (Metrohm, Cheshire, UK). For analysis, screen printed electrodes (SPE) were used (DropSens C223AT). These electrodes consist of working (1.6 mm diameter) and counter electrodes made of gold, while the reference electrode was replaced with an external Hg/Hg_2_SO_4_ (K_2_SO_4_ sat.) (IJ Cambria Scientific, Wales, UK) placed into a salt bridge. Performance of layers generated on SPE were compared with flat gold disc macro working electrodes (1.6 mm diameter, from BASi, Costa Mesa, CA, USA), switched to common three-electrodes cell combined with platinum mesh as a counter electrode and Hg/Hg_2_SO_4_ as reference electrode. All impedances were carried out in 50 mM phosphate buffer pH 7.0, containing 100 mM K_2_SO_4_, in the presence of 2.5 mM/2.5 mM [Fe(CN)_6_]^4−^/[Fe(CN)_6_]^3−^ redox probe and were obtained at 0.2 V *vs.* Hg/Hg_2_SO_4_ with an AC potential of 10 mV amplitude in the frequency range from 100 kHz to 0.1 Hz.

*Contact angle* measurements were performed using an in-house built optical angle measurement system. The setup consisted of 4 parts: a viewing system (camera), a stage, a dispensing system (syringe/pipette) and a measurement system to calculate the contact angle. The electrodes (both flat and screen printed) were placed on the stage and a 10 µL drop was dispensed on the electrode with the dispensing system. The wetting of surface was then captured using a Nikon p520 camera. Contact angle was measured using a screen protractor version 4.0 procured from Iconico. See Figures S1 and S2 in Supporting Information for a block diagram of the experimental setup and typical images, respectively.

### 2.3. Preparation of Ternary Self-Assembled Monolayer Based on Thrombin Aptamers

Prior to use, screen printed electrodes were washed with acetone for 2 min, followed by sonication in ethanol (2 min) and ultra-pure water (2 min).

Gold disc working electrodes with a diameter of 1.6 mm were polished with 50 nm aluminium oxide particles (Buehler, Lake Bluff, IL, USA) on a polishing pad (Buehler), followed by sonication in ethanol, polishing on a blank polishing pad, and sonication in ethanol to remove any particles. Electrodes were subsequently electrochemically cleaned in 0.5 M H_2_SO_4_ by scanning the potential between the oxidation and reduction of gold, −0.05 V and +1.1 V *versus* an Hg/Hg_2_SO_4_ reference electrode, for 50 cycles until there was no further change in the voltammogram.

Prior to immobilisation, DNA aptamer solutions were incubated at 90 °C in a water bath and then exposed to ice cooling to achieve the correctly unfolded structure. Electrodes were rinsed with deionised water, dried in a stream of nitrogen, and exposed to 6 μL of mixed DNA/HDT immobilisation solution (of variable concentration ratios) for 16 h in a humidity chamber. The DNA immobilisation buffer consisted of 50 mM phosphate buffer (PB), 100 mM K_2_SO_4_, 1 mM ethylene diamine tetraacetic acid (EDTA) pH 7.6 and 10% of ethanol being used to solubilise HDT. After immobilisation, electrodes were rinsed in ultra-pure water. To ensure complete thiol coverage of the gold surface, the electrodes were backfilled with 6-mercapto-1-hexanol (MCH) by dropping 6 μL of 1 mM MCH in PB buffer pH 7.0 containing 100 mM K_2_SO_4_ and 10% of ethanol during 50 min. Electrodes were then rinsed with ultra-pure water and stabilised in PB buffer pH 7.0 for 2 h.

### 2.4. Detection of Thrombin Protein and Non-Specific Interactions

Electrodes with immobilised DNA aptamer/HDT/MCH were incubated with different concentrations (1, 10, 100 pM, 1, 10, 25, 50, 100, 250, 500 nM, and 1 μM) of human thrombin diluted in PB buffer pH 7.0 containing 100 mM K_2_SO_4_. Electrodes were then rinsed with the same buffer and EIS measurements were performed. In this work, we studied EIS detection of human thrombin by successive incubation of the biosensor with each solution of Th as well as detection of different Th concentrations on freshly prepared surfaces.

## 3. Results and Discussion

### 3.1. Ternary SAM Layer for Anti-Fouling Aptasensor Properties

Based on the procedure of Campuzano *et al.* [35,36], we studied the immobilisation of thrombin DNA aptamers (ThA) with co-immobilisation of the other components of the ternary SAM layer onto screen-printed electrodes. In Campuzano’s work the ternary SAM layer was composed of 1,6-hexanedithiol (HDT), 1-mercapto-6-hexanol (MCH) and thiolated single-stranded DNA probes for specific detection of DNA target. In that approach, the DNA probe was immobilised on the surface from the mixture with 1,6-hexanedithiol. The use of HDT, which forms hydrophobic alkyl chain bridges on the electrode surface, was effective in reducing non-specific interactions when compared with a common DNA/MCH surface. The ratio of HDT and thiolated DNA probe was optimised and found to behave most effectively at a ratio of 300/0.05 μM (HDT to DNA). To saturate all non-bonded surface sites, 1 mM of 1-mercapto-6-hexanol was used. The composition of such layer was confirmed by studying the charge transfer resistance and capacitance parameters on different steps of preparation. The authors showed that this DNA sensor allows obtaining high specificity of detection, even in clinical samples.

**Figure 1:**
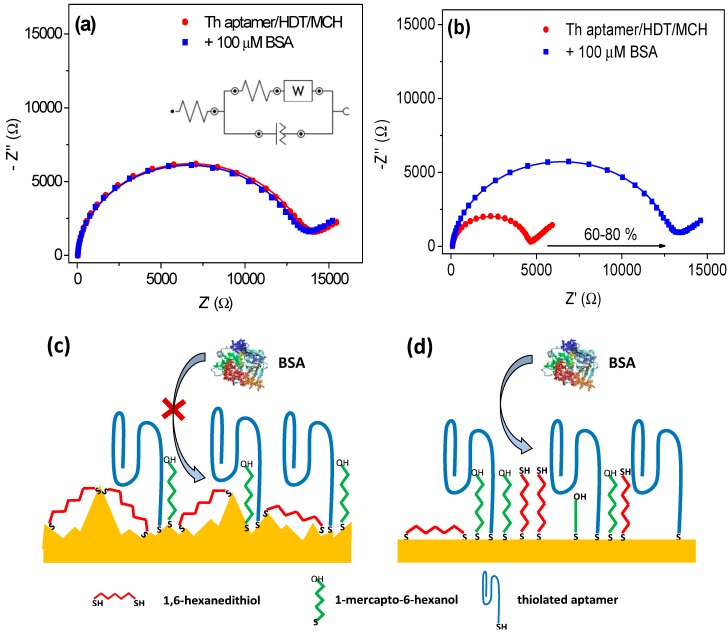
Nyquist plots showing electrochemical impedance spectroscopy responses before (red) and after (blue) incubation of 100 μM bovine serum albumin (BSA) for 30 min on biosensor based on (**a**) screen-printed electrodes (**b**) macroelectrodes modified with thrombin aptamer/ternary self-assembled monolayer 0.05/300 μM and saturated with 1 mM mercaptohexanol. Points show experimental results and solid plots show fitting. Scheme of the surface architecture for (**c**) screen-printed electrodes and (**d**) macroelectrodes.

The thrombin specific aptamer used in this work is a well characterised short DNA sequence (15 bases) that can form a characteristic quadruplex structure. For preparation of the aptasensor using ThA we used a similar approach to Campuzano *et al.*: ThA were co-immobilised with HDT in the ratio of 0.05/300 μM, and then any exposed gold surface was saturated with 1 mM MCH. Construction of biosensor was performed on SPEs. The presence of aptamers was confirmed by comparing such layer with free-aptamers surface using Electrochemical Impedance Spectroscopy (see Supporting Information Figure S3). In order to test the anti-fouling properties of the ternary SAM, electrodes were incubated with high concentration (100 μM) Bovine Serum Albumin solution. The response of sensor was then monitored by EIS in the presence of 2.5 mM of the redox species [Fe(CN)_6_]^3−/4−^. Figure 1a shows the Nyquist plots obtained before and after incubation of the biosensor with BSA solution. The data were fitted using a Randles equivalent circuit model (see inset Figure 1a), where *R*_s_ is the electrolyte resistance and *R*_ct_ is the electron transfer resistance; the double layer capacitance was replaced by a constant phase element (CPE), which accounts for irregularities on the surface due to roughness of the gold substrate and inhomogeneities on the biolayer; a Warburg element was added to account for diffusion processes of the redox marker. The fitting results show a very slight variation in the *R*_ct_ value, from 14.1 kΩ to 13.8 kΩ after BSA incubation, *i.e.*, a −1.5% of relative change Δ*R*_ct_/*R*_ct0_, where Δ*R*_ct_ = *R*_ct_ – *R*_ct0_; *R*_ct0_ and *R*_ct_ are the charge transfer resistance obtained, respectively, before and after incubation of the aptasensor with protein, respectively. The same behaviour was obtained when ThA was replaced with an aptamer specific for streptavidin (SA) of higher molecular weight (60 bases) and which are characterised by forming a secondary “loop” structure (see Supporting Information, Figure S4). The SAMs appeared very resistant to antifouling and based on the work of Campuzano *et al.* where detection in urine and serum samples was successfully performed, application of aptasensors based on a ternary SAM layer for clinical samples is promising.

Results obtained on SPEs were compared with standard three-electrode cell with gold disc as working electrode (macroelectrode), platinum mesh as counter electrode and Hg/HgSO_4_ as reference electrode. After incubation of the electrode with 100 μM BSA, a substantial variation (60%–80%) in *R*_ct_ was observed. These seemingly contradictory results could be explained by taking into account the different surface architectures of the SPEs in comparison to polished macroelectrodes. The topology of SPEs is highly rough and heterogeneous which favours the formation of bridges by 1,6-hexadithiol [37], while the macroelectrode surface is considerably flatter and may impede the attachment of HDT to the surface by both thiol groups, favouring the formation of a compact HDT layer with exposed thiol groups. Such planar surface with compact HDT layer was characterized by Morel *et al.* [38], where unbound thiol groups were used for attachment of gold nanoparticles for biosensor construction. The schematic explanation of the difference between SPEs and macroelectrode is presented in Figure 1c,d.

**Figure 2:**
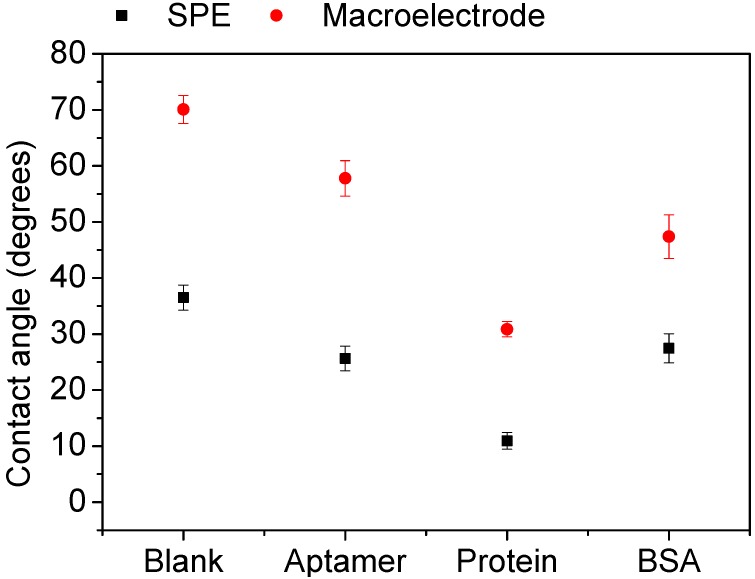
Contact angle values measured for screen-printed electrodes (SPEs) and polished gold surfaces on the different steps of aptasensor construction. All measurements were repeated on three separate samples.

The results were confirmed by measuring the contact angle of the aptasensors constructed on SPEs and polished gold surfaces. The contact angle was determined on blank un-modified surface, after SAM layer preparation and after incubation with specific thrombin protein or non-specific BSA solution. Figure 2 presents the obtained results (see Figure S2 in Supplementary Information for the contact angle images). The contact angle of blank SPEs is 36.5° and decreases after SAM immobilisation and after subsequent interaction with Th protein (respectively, 25.6° and 10.9°). After incubation of the aptasensor with BSA instead of Th solution, the value remains almost unchanged (27.4°), which confirms the anti-fouling properties of the SAM. The polished gold electrode has a similar behaviour after SAM layer immobilisation and Th incubation (the values decrease from 70.0° to 57.7° and 30.8°, respectively). However after exposure to non-specific BSA solution, a high variation is observed (47.4°), which suggests that the topology of the electrode surface is crucial for anti-fouling properties of aptasensors based on ternary SAM layers and confirms the results obtained by EIS.

### 3.2. Optimisation of the Ratio Aptamer/Hexanedithiol

**Figure 3:**
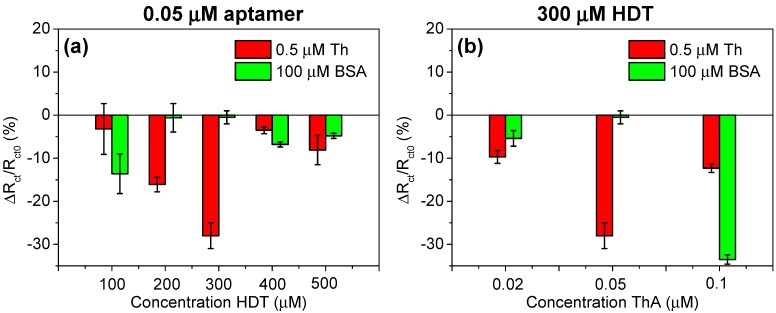
Charge transfer resistance (*R*_ct_) values variation obtained for incubation of thrombin aptamer (ThA) sensors with 0.5 μM of thrombin (Th, red bars) and 100 μM of bovine serum albumin (BSA, green bars). Figures represent the results obtained for different ratios of aptamer/hexanedithiol (HDT), for (**a**) 0.05 μM ThA and (**b**) 300 μM HDT.

Aptamers, contrary to DNA probes, can form secondary (“loops”) and three-dimensional (quadruplex) structures, which may affect analytical performances as well as anti-fouling properties of biosensors based on ternary SAM layers. Therefore some optimisation in their construction should be performed to ensure that the optimised conditions previously reported for DNA hybridisation, are also valid for DNA aptamers. In particular the ratio between aptamers and HDT can have an important effect on the ability of the DNA aptamers to form a secondary structure and bind to its target protein. Varied aptamer concentrations (0.02, 0.05, and 0.1 μM) and HDT concentrations (100, 200, 300, 400, and 500 μM), all backfilled with 1 mM of MCH were tested for the construction of the aptasensors. These biolayers were incubated with 0.5 μM of human thrombin or 100 μM BSA to verify specific as well as non-specific interactions. Figure 3 shows the values obtained for the relative variations of charge transfer resistance (Δ*R*_ct_/*R*_ct0_) in the case of specific interactions with Th (red bars) and non-specific interactions with BSA (green bars). The interactions of ThA with Th result in decreasing of the *R*_ct_ value and depend on the quantity of aptamer immobilised on the surface. The highest *R*_ct_ variation was observed for 0.05/300 μM aptamer/HDT ratio (−28%) and optimal signal-to-noise ratio was obtained for 200–300 μM HDT (Figure 3a). However, variation of aptamers concentration for optimal 300 μM HDT did support the desired level of anti-fouling properties (Figure 3b). A 0.02 μM concentration of aptamer resulted in lower Th binding (−10%), most likely due to the lower number of bioreceptors on the surface and higher BSA adsorption, possibly due to larger areas containing MCH. Conversely, increasing of quantity of aptamers on the electrode surface (0.1 μM) can cause steric hindrance effects for significant thrombin attachment (−12%) and insufficient saturation of the surface with HDT giving high *R*_ct_ value variation after incubation of biosensor with BSA (−33%).

To improve anti-fouling properties on the polished gold surface, similar optimisations were also performed. Different concentrations of HDT in the range 200–400 μM were used but without any improvement of non-specific binding.

Based on these results, the ThA aptamers should be used in 0.05 μM concentration with optimal 300 μM HDT ratio allowing avoid non-specific interactions, in a similar way as it was reported by Campuzano *et al.* [35,36] for DNA sensing.

### 3.3. Analytical Performances of Aptasensors Based on Ternary SAM Layer

**Figure 4:**
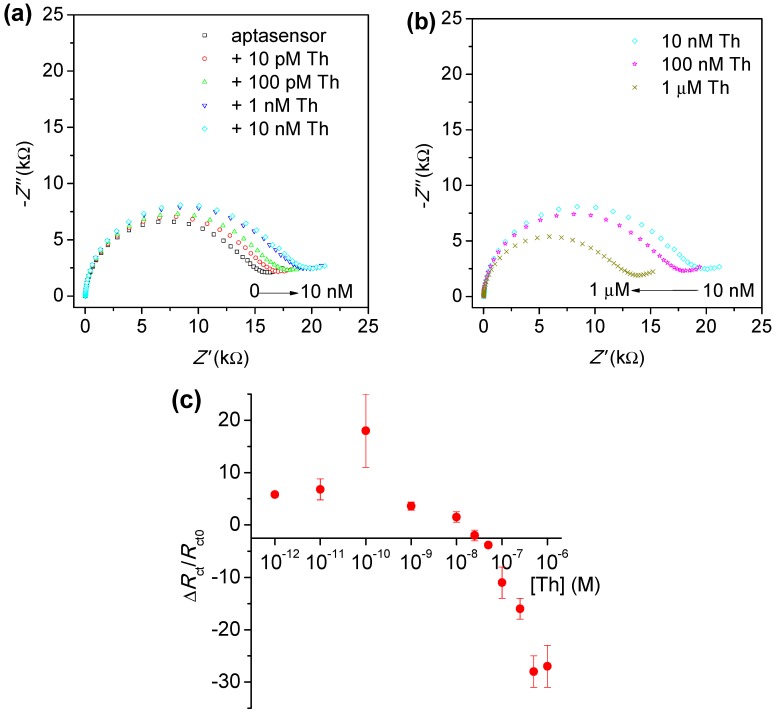
Nyquist plots obtained after successive incubation of biosensors with thrombin in concentrations range (**a**) from 10 pM to 10 nM and (**b**) from 10 nM to 1 μM. (**c**) Calibration curve obtained based on charge transfer resistance (*R*_ct_) value variation after incubation of freshly prepared biosensors with various thrombin (Th) concentrations. The error bars represent standard deviation for three independents measurements.

SPEs modified with ThA aptamer/HDT in the optimised ratio of 0.05/300 μM and saturated with 1 mM MCH were used for detection of Th in a wide range of concentrations in order to determine the sensitivity of the biosensor. The stability of the biosensor was verified by repeating EIS measurements every 30 min and immersing the electrodes in measurement buffer free from proteins in-between measurements (see Supporting Information, Figure S5). Then, biosensor was incubated successively in solutions of Th at: 10, 100 pM, 1, 10, 100 nM and 1 μM. After each incubation, the electrode was analysed using EIS in the presence of 2.5 mM [Fe(CN)_6_]^3−/4−^ redox probe (Figure 4a,b). Incubation of biosensor in the range of concentrations 10 pM–10 nM resulted in successive increases of the *R*_ct_ value (Figure 4a). However, higher concentrations of Th such as 100 nM and 1 μM conversely caused a substantial decrease in the *R*_ct_ value (−11% and −27%, respectively). This behaviour could occur from some non-specific attachment of Th to the electrode surface at saturating levels. To test this observation further, successive detection of thrombin in PB buffer containing 0.05% Tween was performed; however, the same behaviour of increasing and then decreasing of *R*_ct_ with increasing Th concentration was obtained (see Supplementary Information, Figure S6).

These results were therefore compared with detection of Th protein where each concentration was incubated on separate, freshly prepared sensors. Figure 4c presents the relative changes of charge transfer resistance obtained during detection of various concentrations of Th protein on these aptasensors. As with the data with successive increases in Th concentration, for lower range of Th (1–100 pM) an increase in *R*_ct_ was observed. But with the highest concentrations this increasing was less substantial and finally from 25 nM a decrease in *R*_ct_ was obtained with saturation reached for 1 μM.

**Figure 5:**
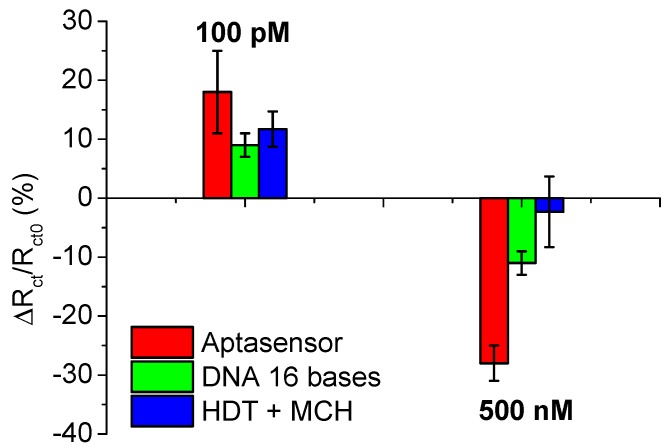
Interactions of thrombin in concentrations of 100 pM or 0.5 μM with screen-printed electrodes surfaces modified with thrombin aptamers/hexanedithiol (HDT) and backfilled with mercaptohexanol (MCH, red bars), DNA of 16 bases sequence co-immobilised with MCH (green bars) and HDT backfilled with MCH (blue bars).

These results supported further investigation into the potential causes of the non-specific interactions of Th with the surface and find the cause of different behaviour of *R*_ct_ value variation depending on the Th concentration. To do this, Th protein (at concentrations of 100 pM and 0.5 μM) was incubated on surfaces functionalised with either ThA (Figure 5, red bars) or surfaces modified with thiolated DNA of the same length as the ThA aptamer, but of a randomised sequence (Figure 5, green bars). Additional surfaces co-immobilised with only HDT backfilled with MCH (Figure 5, blue bars) were also tested. Figure 5 shows that the variation of *R*_ct_ values after incubation of these three classes of surface with 100 pM of Th is almost the same. However, *R*_ct_ variation after incubation of 0.5 μM of thrombin strongly depends on the surface modification. *R*_ct_ variation is much higher when the surface is modified with specific aptamer and lowers when the surface contains negatively charged DNA, which can electrostatically, non-specifically interact with protein. On the surface modified just with HDT there was no non-specific interactions.

Using the EIS method for target detection in the presence of [Fe(CN)_6_]^3−/4−^ redox probe, different effects can be observed. Usually detection of hybridisation of DNA probe attached to the surface with DNA target from solution results in an increase of the charge transfer resistance value [39]. This can be due to the negative charge of the DNA probe, which increases after hybridisation. The electronegative phosphate skeletons prevent [Fe(CN)_6_]^3−/4−^ from reaching the electrode surface during the process of redox reaction, which decreases the ability of the electrode surface to transfer electrons resulting in an increase of *R*_ct_. However, when detection concerns proteins using DNA aptamers, two different behaviours can be observed. Binding of proteins can result in decrease [17] or increase [40] in charge transfer resistance which is due to the charge of protein screening the DNA charge or forcing a higher density of DNA charges closer to the electrode upon folding. This can also be applied for the determination of small molecules such as ochratoxin. Detection of this toxin resulted in expansion of semicircle, which was explained by the negative charge of the target molecule [11]. However, another work shows decreasing of *R*_ct_ value due to ochratoxin detection and was presented as a result of changing in DNA aptamers structure after molecule attachment which can facilitate ions permeability.

For thrombin aptamers, an increase in the *R*_ct_ value is usually reported [40,41,42]. In our work, two contrary process could however be observed. At low concentrations of Th, *R*_ct_ increases as expected. This can be explained by the accumulated negative charge of thrombin, which repulses the redox reporter from the biolayer. At higher concentrations a second process seems to predominate, which results in a reduction in *R*_ct._ The second process could potentially concern changes in aptamer structure due to protein attachment. This effect would be likely to have influence on surface architecture, potentially generating spaces within the binding surface that allow the redox probe to more easily penetrate the biolayer thus decreasing *R*_ct_.

However, the EIS method can also have limitations due to false positive signals. These non-specific impedance changes can be due to initial electrode contaminations, repetitive measurements or additional incubations in the buffer between measurements and can be easily confused with a specific impedance signal [43]. In our work, we did not exclude the possibility that the positive variation in charge transfer resistance resulted from a false positive signal.

### 3.4. Influence of pH and Temperature on Thrombin Detection

**Figure 6:**
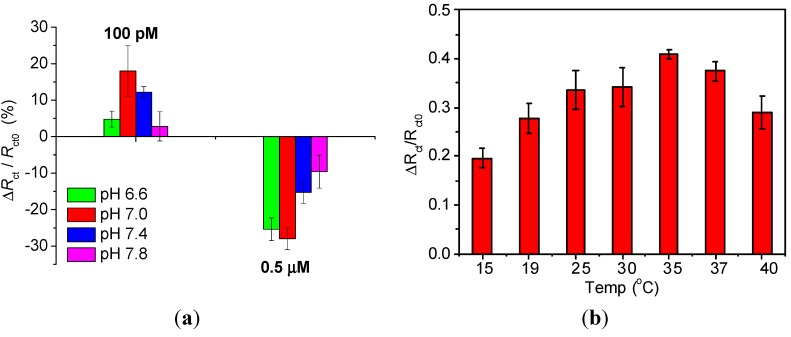
(**a**) Influence of buffer pH on electrochemical response of biosensor; influence of buffer pH was studied by interactions of aptasensor with two thrombin concentrations 100 pM and 0.5 μM. (**b**) Influence of incubation temperature on biosensor response studied by incubation of aptasensor with 0.5 μM thrombin.

pH variations can significantly affect the aptasensor’s response due to changes of the charge of Th as well as screening of the DNA aptamer charge. Therefore, we studied the influence of different pH on the aptasensor response after interaction with 100 pM or 0.5 μM Th (Figure 6a). Measurements and incubation of biosensor with thrombin protein was performed in PB buffers with pH 6.6, 7.0, 7.4 and 7.8. Interactions of aptamer/thrombin for a concentration of 100 pM resulted in positive changes in charge transfer resistance for each of the pH values studied, while after incubation of aptasensor with 0.5 μM Th a decrease was observed. At pH 6.6 the interaction of aptasensor with 100 pM of Th is very low, but high *R*_ct_ variation was observed after incubation with 0.5 μM of Th (green bars). The highest interaction with 100 pM or 0.5 μM of Th was observed at pH 7.0 (red bars). Increasing of buffer pH caused lower variation in charge transfer resistance after interactions with these two different Th concentrations.

Further studies concerned the influence of temperature during incubation with specific target on aptasensors response. Freshly prepared aptasensors based on ThA aptamer/ternary SAM were incubated with 0.5 μM of human thrombin at 15, 19, 25, 30, 35, 37 or 40 °C. After incubation of aptasensor with human thrombin in each temperature, a decrease in R_ct_ value was observed. Figure 6b presents the variation of *R*_ct_ values after incubation of aptasensor with Th depending for different temperatures. The best biosensor response was observed for a temperature of 35 °C, which can signify the highest aptamer/protein interactions. However, with further increasing of the temperature the biosensor response resulted in lower aptamer/protein interactions, which might be due to denaturation or unfolding of the DNA aptamers or destabilisation of the underlying thiol immobilised SAM layer. The influence of pH and temperature on biosensors’ response is important to study as ternary SAM platforms can be generalized for detection in different complex matrices such as serum, waste water or bacterial cultures, for example. This can be obtained by immobilisation of other specific aptamers for detection of various molecules such as drugs, toxins, microorganisms, *etc.*

### 3.5. Stability of Aptasensor Based on Ternary SAM Layer

**Figure 7:**
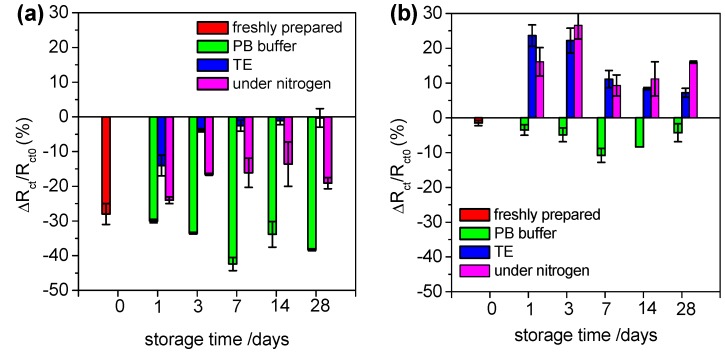
Influence of storage time and buffers on (**a**) interactions aptamers/thrombin by incubation of biosensor with 0.5 μM thrombin (Th) and (**b**) anti-fouling properties of by incubation of biosensor with 100 μM bovine serum albumin (BSA). Results were obtained by storage of biosensors in PB buffer (green bars), TE buffer (blue bars) and under nitrogen conditions (violet bars) and compared with *R*_ct_ values variation after incubation of freshly prepared biosensor with Th or BSA (red bars).

The stability of the aptasensor based on the ternary SAM layer was also studied. For this purpose aptasensors were stored at 4 °C in PB buffer, in Tris/EDTA (TE) buffer or under nitrogen conditions for 1, 3, 7, 14 and 28 days after preparation. Then performance and anti-fouling properties of such biosensors were verified by detection of human thrombin (0.5 μM) or non-specific adsorption of BSA (100 μM), respectively. The relative changes of *R*_ct_ values after Th detection for different storage conditions as a function of time storage are compared on Figure 7a. Figure 7a shows variation in *R*_ct_ after incubation of aptasensor stored in PB buffer (green bars), Tris buffer (blue bars) and under nitrogen (pink bars) with human thrombin. Red bars represent the response of freshly prepared biosensor. The highest performance of biosensor was observed when the sensor was stored in PB buffer, where interactions aptamer/thrombin was observed even 28 days after preparation. Storage in TE buffer allows detection of protein only on freshly prepared aptasensors.

Influence of storage time and buffers on anti-fouling properties of biosensor is presented on Figure 7b. Using of PB buffer allows avoiding high non-specific interactions, while storage in other conditions resulted in important non-specific interactions. Even though these non-specific interactions in TE buffer and in nitrogen gas could be distinguished from specific responses of the biosensor due to the signal of the *R*_ct_ variation, the detection of thrombin in complex samples would be severely compromised. For storage in PB buffer, on the other hand, the data clearly indicates that the sensors retain their sensitivity and anti-fouling properties when stored for up to 28 days.

## 4. Conclusions

We have demonstrated that antifouling properties of aptasensors (using a thrombin aptasensor as a case study) can be achieved by means of a ternary SAM layer with the same conditions as those reported for DNA hybridisation sensors [35]. However, one of the key aspects of this work is the observation that, although the ternary SAM structure provides good antifouling properties when used on screen-printed electrodes, it is rather inefficient when smoother gold surfaces are employed, presumably due to the orientation of the hexanedithiol molecules on the surface.

For thrombin aptasensors, conflicting variations in charge transfer resistance are observed: An increase in *R*_ct_ is observed for low concentrations of thrombin target, while at higher concentrations a negative shift is observed. Through the controls used, we speculate that the positive shifts at low concentrations are due to non-specific interactions of thrombin with DNA, which reach saturation for concentrations higher than 100 pM, while the negative shifts observed at higher concentrations are due to specific binding of the thrombin to the DNA aptamer.

The pH and temperature of the buffer used during incubation of the sensor also strongly affect the performance of the aptasensors. Optimised binding was observed for pH 7.0 and 35 °C. Stability of the aptasensors was studied for different storage conditions and a shelf life of at least 28 days was determined when the sensors are stored in phosphate buffer.

The results show that great care needs to be given to the characterisation of any impedance-based aptasensor. Surface chemistries to immobilise aptamers and provide antifouling properties need to be optimised for each aptasensor taking into account the type of electrode used, including its roughness. Factors such as buffer, pH, temperature and storage conditions also strongly influence the performance of the sensors. A full dose response of the sensors is required from very low to very high concentrations of the target, as well as the use of suitable controls, to ensure that the target is binding specifically to the DNA aptamer. Upon a full characterisation of the aptasensors, their application on clinical or environmental samples can be envisaged using ternary SAM layers and controlled measurement conditions.

## Supplementary Files

Supplementary File 1
