# Supplementary material for: Optimisation and Characterisation of Anti-Fouling Ternary SAM Layers for Impedance-Based Aptasensors
Source: Sensors (Basel). 2015 Sep 29;15(10):25015–32. doi: 10.3390/s151025015 (PMC4634408; doi:10.3390/s151025015)
Supplement: Supplementary File 1 [file sensors-15-25015-s001.pdf]

OPEN ACCESS

Sensors

ISSN 1424-8220

www.mdpi.com/journal/sensors

Supplementary Information

Optimisation and Characterisation of Anti-Fouling Ternary SAM Layers for Impedance-Based Aptasensors. *Sensors* 2015, *15*, 25015-25032

Anna Miodek [1,*], Edward M. Regan [1,†], Nikhil Bhalla [1], Neal A.E. Hopkins [2], Sarah A. Goodchild [2] and Pedro Estrela [1,*]

[1] Department of Electronic and Electrical Engineering, University of Bath, Bath BA2 7AY, UK; E-Mails: ed_regan@hotmail.com (E.M.R.); n.bhalla@bath.ac.uk (N.B.)

[2] Defence Science and Technology Laboratory, Porton Down, Salisbury, SP4 0JQ, UK; E-Mails: nahopkins@dstl.gov.uk (N.A.E.H.); sagoodchild@dstl.gov.uk (S.A.G.)

† Current address: Clarity BioSolutions Ltd., Building 114, Porton Down Science Park, Salisbury, SP4 0JQ, UK

* Authors to whom correspondence should be addressed; E-Mails: a.miodek@bath.ac.uk (A.M.); p.estrela@bath.ac.uk (P.E.); Tel.: +44-1225-386324 (P.E.); Fax: +44-1225-386305 (P.E.).

1. Contact Angle Measurements

A scheme of the contact angle setup used is shown in Figure S1. Contact angle measurements were performed on both screen-printed electrodes and polished gold electrodes after each immobilisation step (Figure S2).

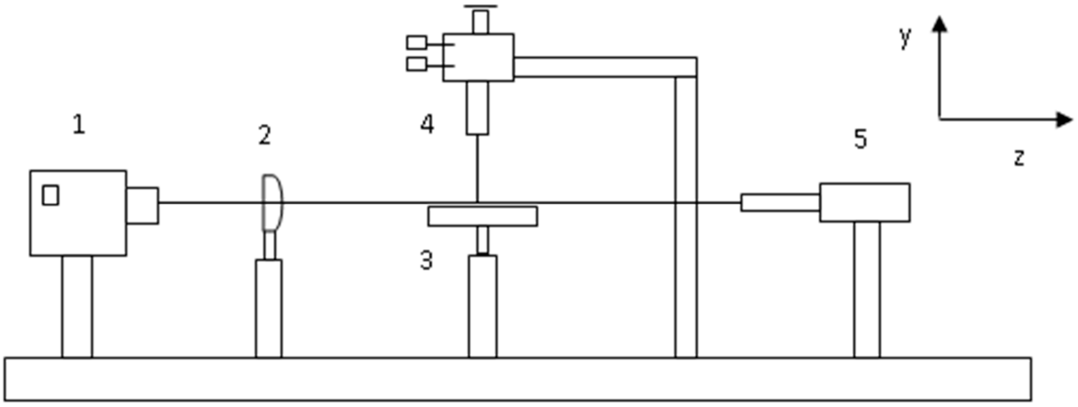

Figure S1. Block diagram of experimental setup. 1: Nikon Bridge camera P520, 2: Thorlabs lens, 3: Drop stage, 4: Hamilton syringe, 5: Nicholas illumination.

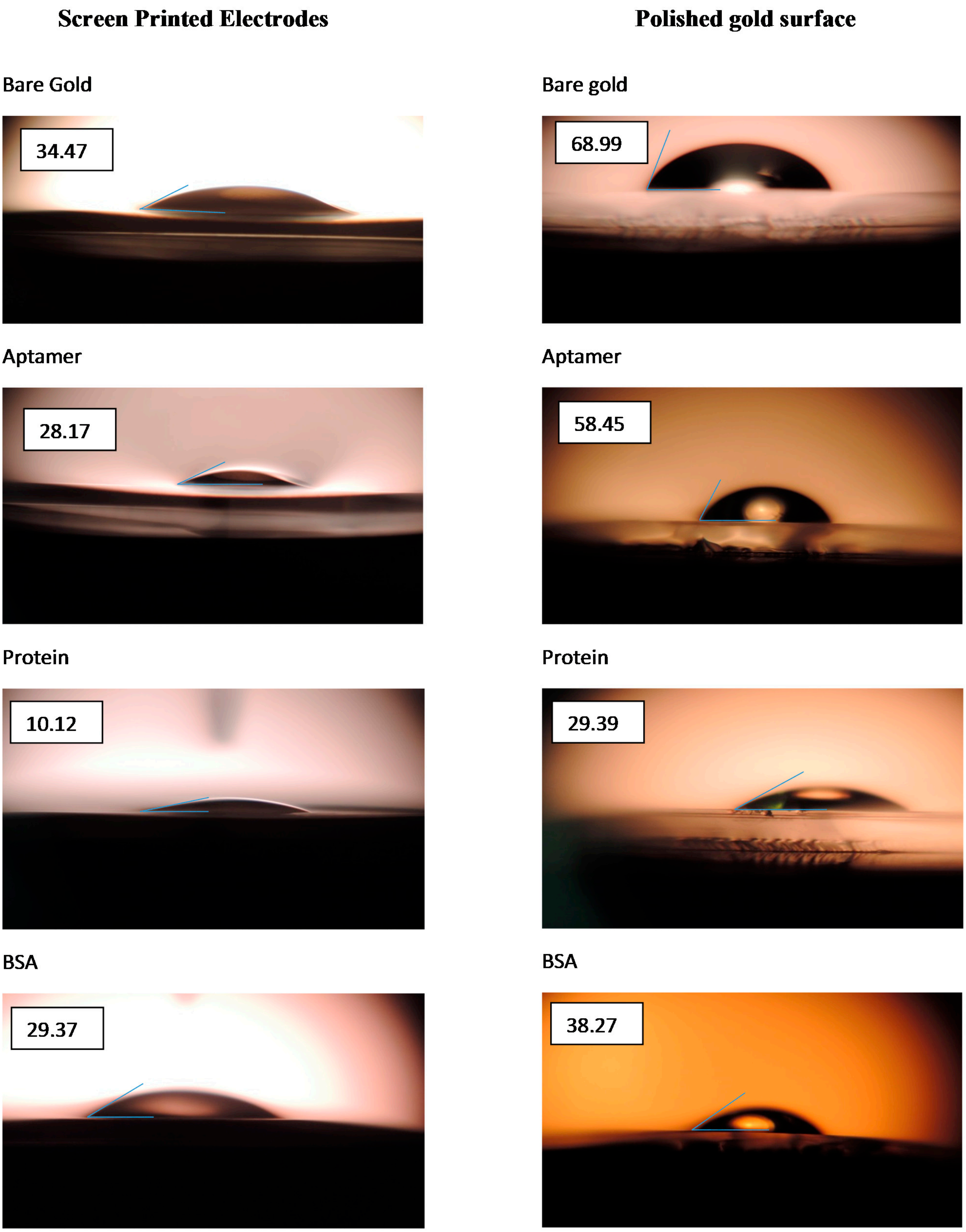

Figure S2. Contact angle measured for aptasensors prepared on screen-printed electrodes and polished gold electrodes at different steps of construction.

2. Characterisation of Ternary SAM Layer

The ternary SAM layer was characterised by means of electrochemical impedance spectroscopy (EIS) and compared to layers only comprising HDT and MCH to show the binding of the aptamer (Figure S3).

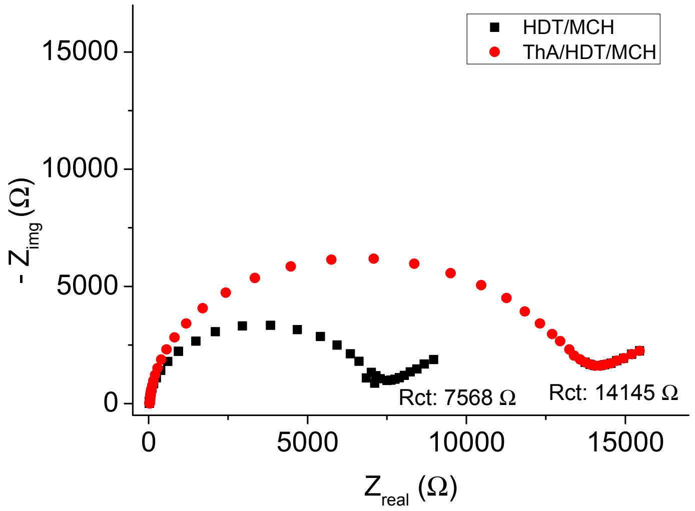

Figure S3. Nyquist plots showing electrochemical impedance spectroscopy responses for ternary self-assembled monolayer of 0.05 μM/300 μM aptamer/hexanedithiol (HDT) (red) and for aptamers-free surface composed of HDT and mercaptohexanol (MCH) (black).

3. Anti-Fouling Properties of Biosensor Based on Streptavidin Aptamers

Figure S4 shows the anti-fouling properties of the ternary SAM layer against BSA.

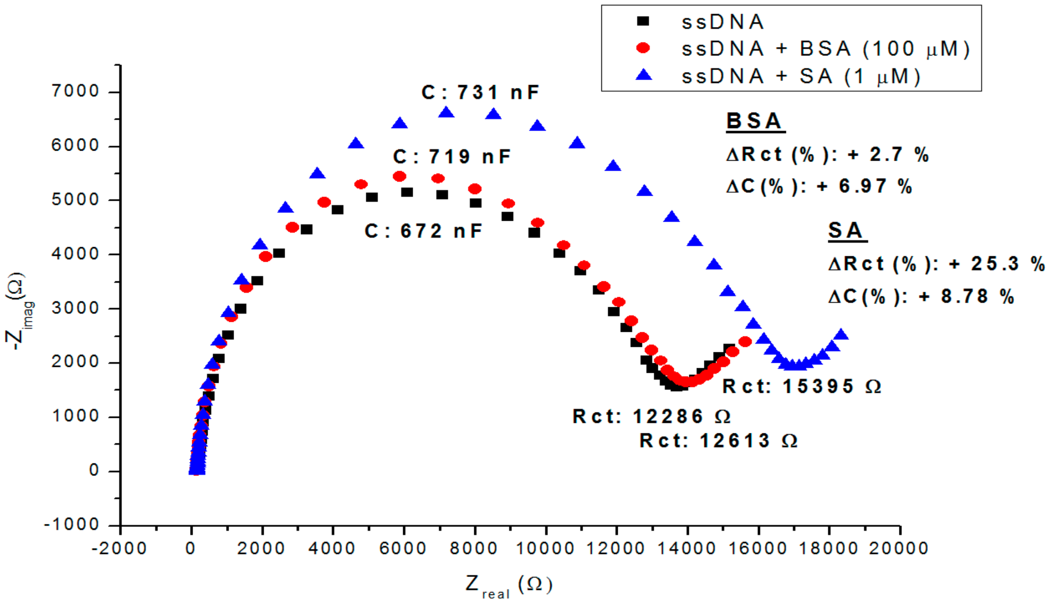

Figure S4. Nyquist plots showing electrochemical impedance spectroscopy responses before (black) and after incubation of 100 μM of bovine serum albumin (BSA) (red) or 1 μM of streptavidin (blue) for 30 min on biosensor based on 0.05 μM/300 μM streptavidin aptamer/hexanedithiol ternary self-assembled monolayer.

4. Detection of Thrombin from Human Serum and Studies on False Positive Signal

The stability of the ternary SAM aptasensor is shown in Figure S5, while Figure S5 shows the effect of the addition of Tween 20 to remove non-specifically bound thrombin.

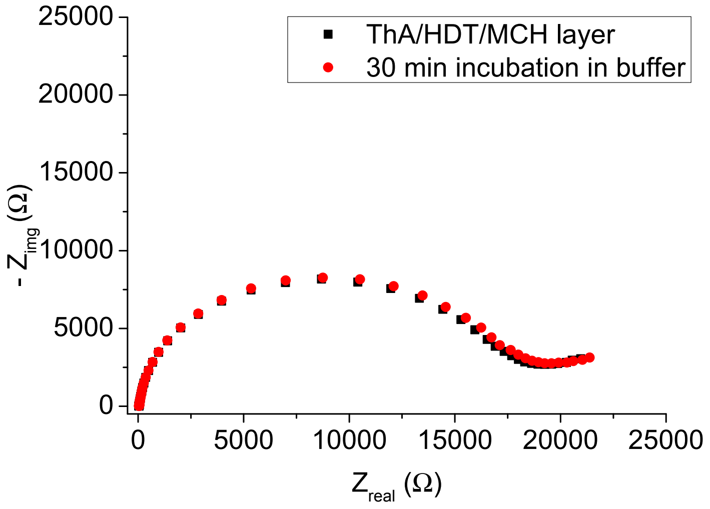

Figure S5. Nyquist plots present stability of aptasensor before (black) and after incubation in measurement buffer for 30 min. The variation of R_{ct} value was calculated as 1.6 ± 1%, based on 10 independent measurements.

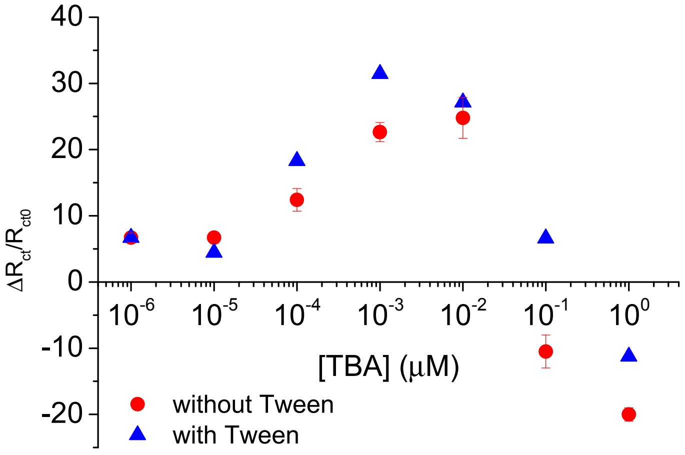

Figure S6. Detection of various concentrations (1 pM–1 µM) of human thrombin in phosphate buffer with 0.1 M K_2SO_4 containing Tween 20 (blue points) or not containing Tween 20 (red points).

© 2015 by the authors; licensee MDPI, Basel, Switzerland. This article is an open access article distributed under the terms and conditions of the Creative Commons Attribution license (http://creativecommons.org/licenses/by/4.0/).
